# Tridimensional cell culture of dermal fibroblasts promotes exosome-mediated secretion of extracellular matrix proteins

**DOI:** 10.1038/s41598-022-23433-0

**Published:** 2022-11-17

**Authors:** Vincent Clément, Vincent Roy, Bastien Paré, Cassandra R. Goulet, Lydia Touzel Deschênes, François Berthod, Stéphane Bolduc, François Gros-Louis

**Affiliations:** 1 Department of Surgery, Faculty of Medicine, Laval University, Québec, QC Canada; 2 Division of Regenerative Medicine, Laval University Experimental Organogenesis Research Center/LOEX, CHU de Québec Research Center – Enfant-Jésus Hospital, Québec, QC Canada

**Keywords:** Biological techniques, Cell biology

## Abstract

Extracellular matrix (ECM) secretion, deposition and assembly are part of a whole complex biological process influencing the microenvironment and other cellular behaviors. Emerging evidence is attributing a significant role to extracellular vesicles (EVs) and exosomes in a plethora of ECM-associated functions, but the role of dermal fibroblast-derived EVs in paracrine signalling is yet unclear. Herein, we investigated the effect of exosomes isolated from stimulated human dermal fibroblasts. We report that tridimensional (3D) cell culture of dermal fibroblasts promotes secretion of exosomes carrying a large quantity of proteins involved in the formation, organisation and remodelling of the ECM. In our 3D model, gene expression was highly modulated and linked to ECM, cellular migration and proliferation, as well as inflammatory response. Mass spectrometry analysis of exosomal proteins, isolated from 3D cultured fibroblast-conditioned media, revealed ECM protein enrichment, of which many were associated with the matrisome. We also show that the cytokine interleukin 6 (IL-6) is predicted to be central to the signalling pathways related to ECM formation and contributing to cell migration and proliferation. Overall, our data suggest that dermal fibroblast-derived EVs participate in many steps of the establishment of dermis’s ECM.

## Introduction

The extracellular matrix (ECM) tightly controls cell fate in physiological and pathological processes^1,2^. Consisting of a variety of proteins and polysaccharides that are secreted and assembled locally, the ECM also provides biomechanical and biochemical support to cells, and orchestrates the architectural framework of stromal tissues^3–5^. The composition, assembly and physical properties of the ECM critically influence cell signalling pathways and affect cell functions such as adhesion, migration, proliferation, polarity, shape, differentiation, and apoptosis^6^. In a living multicellular organism, the secretory pathway responds to constant demands for cargo delivery during development, physiological changes and tissue repair. Although evidence show that ECM deposition pathway relies on functional core secretion machinery, the molecular mechanisms by which fibroblasts regulate ECM secretion remain poorly understood^7^.

Extracellular vesicles (EVs) are detected in most biologic fluids such as saliva, plasma and cerebrospinal fluid^8,9^, and can be released by different cell types^10,11^. Exosomes, a subgroup of EVs with a highly variable size of 30 to 100–150 nm in diameter, are composed of a lipid bilayered membrane containing biologically active molecules such as proteins, lipids, carbohydrates and nucleic acids—including DNA, coding and non-coding RNAs^10,12^. Depending on their cellular origin, exosomes contain specific profiles of cellular proteins, signalling proteins and/or peptides, and have been shown to regulate diverse physiologic and pathologic processes, such as angiogenesis, cell differentiation and fate, epithelial to mesenchymal transition, and apoptosis^13^. Since exosomes allow donor cells to modulate the behaviour of recipient cells through the transfer of cargo, they have emerged as mediators of a newly discovered cellular paracrine communication^14^. Recent findings have also established exosomes as key players in tissue injury repair^15,16^.

Exosomes contain a plethora of components, including integrins, matrix remodelling enzymes and members of the immunoglobin superfamily, which are capable of directly interacting with the ECM^17,18^. Moreover, the presence of ECM-associated enzymes, such as matrix metalloproteinases (MMPs), heparanases, hyaluronidases, extracellular matrix metalloproteinase inducer, adamalysin metalloproteinases and tissue inhibitors of metalloproteinases (TIMPs) in exosomes have been shown to modulate directly the structural architecture and dynamics of the matrix^19–23^. Accumulating evidence indicates an essential role of EVs secreted by dermal fibroblasts in various biological processes involving ECM components^24–27^. Despite these observations, the complex roles of fibroblast-derived exosomes in molecular mechanisms that underlie dermal ECM secretion, deposition, assembly and remodelling, still remain largely unknown.

In the present study, we report that tridimensional (3D) cell culture affects exosomal content of dermal fibroblasts, in which proteins are actively participating to ECM establishment and stability. Using a scaffold-free 3D fibroblast culture system, based on tissue engineering approaches, we show that ECM components are secreted in the intercellular space via exosome secretion pathways. We also show that the isolated exosomes properly promote cellular migration and proliferation, partly through IL-6 signalling. Our data demonstrate that fibroblasts-derived exosomes and possibly other EVs might have a significant impact on the formation of the dermal matrix.

## Results

### Dermal fibroblasts secrete homogeneously distributed exosomes when cultured in a 3D environment

**Figure 1:**
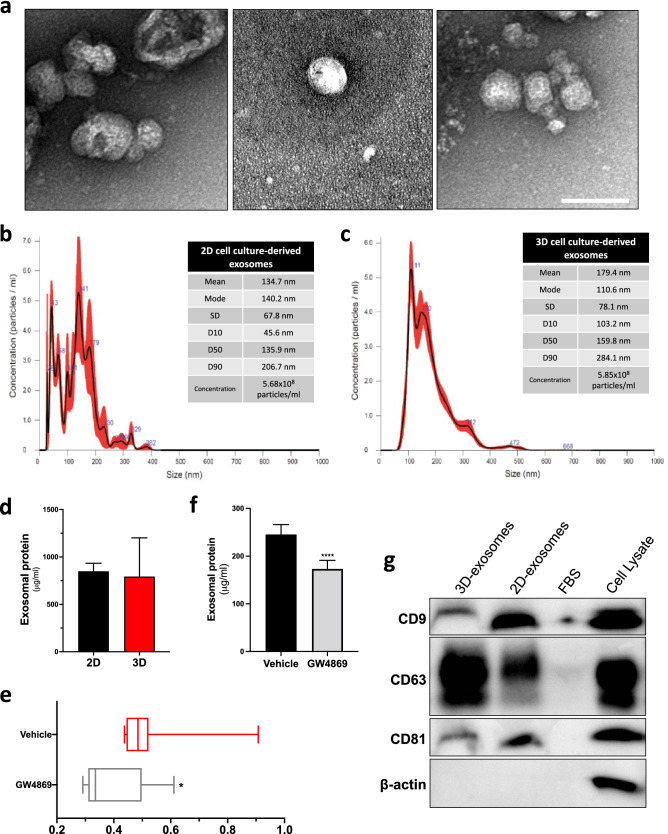
Dermal fibroblasts cultured in 3D secrete exosomes with a more homogenous size distribution. Transmitted electron microscopy images of exosomes isolated from dermal fibroblasts cultured in 3D (**a**). Scale bar = 100 nm. Particle’s size distribution analysis of exosomes isolated from conditioned media of dermal fibroblasts cultured in 2D (**b**) and in 3D (**c**). 6 exosomal populations were pooled for the analysis and samples were injected 3 to 5 times. Exosomal protein concentration according to the cell culture method (**d**). Concentrations were reported in supernatant volume (mL) and normalised with the number of cells. *N* = 6; *n* = 2. Acetylcholinesterase activity quantification of 3D fibroblast culture treated with 30 μM of the exosome secretion inhibitor GW4869 (**e**). *N* = 6; *n* = 2. Exosomal protein concentration of GW4869-treated fibroblasts (**f**). *N* = 2; *n* = 4. Characterization of specific exosomal makers (CD9, CD63 and CD81) of extracellular vesicles depending on the fibroblasts culture method (**g**). β-actin was used as a cellular marker. Note that the cropped images only are shown for conciseness; see supplementary figures section to see the original blots. Statistical analyses were performed by an unpaired T-test with Welch’s correction. Graphs show mean ± SD. *P < 0.05 and ****P < 0.0001.

To assess whether cell culture approaches, monolayered (2D) and 3D, have an influence on exosomes and EVs release and characteristics, we initially analysed the size distribution of the isolated microparticles using a highly precise technology. First, we sought to determine the secreted EVs sizes using different methods. Transmission electron microscopy (TEM) imaging, which is known to be the most accurate, although technically challenging, EV sizing method, revealed close-to-spherical shaped EVs with typical exosomal morphology and sizes lower than 100 nm in diameter (Fig. 1a). Size distribution was also measured by nanoparticle tracking analysis (NTA) measurement with Nanosight NS300. Using this approach, exosome diameter ranges were found to be relatively similar for both cell culture methods (Fig. 1b,c). In fact, 2D culture-derived exosomes measured 134.7 nm ± 67.8 nm, and sizes were distributed from 28 to 383 nm (Fig. 1b), while 3D culture-derived exosomes measured 179.4 nm ± 78.1 nm, and sizes were mostly distributed from 100 to 350 nm (Fig. 1c). Note here that NTA size measurements has been shown to measure substantially larger EV diameters and give broader size distribution when compared to TEM^28^. This discrepancy might at least be due to the fact that part of the EVs are below the resolution limit of NTA. Although 3D-exosomes were also seemingly more homogeneous in diameter compared to 2D-exosomes, the total exosomal protein concentration was similar between conditions following normalisation (Fig. 1d). While significant variations were observed in the expression levels of genes involved in the biogenesis of exosomes, no obvious correlation between exosome biogenesis and the tested cell culture approaches can be drawn (Supplementary Fig. 1).

We then sought to determine if GW4869 treatment, a well-known selective exosome release inhibitor, influenced EVs secretion. Indeed, we observed an attenuation of exosomes released upon GW4869 treatment, measured by exosomal acetylcholinesterase (AChE) activity (*p* < 0.05) (Fig. 1e). Consistent with a decrease in AChE activity, indicative of decreased exosomal secretion^29^, lower exosomal protein concentrations were also measured following treatments with GW4869 (Fig. 1f). Specific exosomal markers expression profiles were then determined by western blot analysis (Fig. 1g and Supplementary Fig. 2). Expression of CD9, CD63 and CD81, which are the well-known tetraspanins expressed on the external exosomal membrane can be observed in both type of tested culture approaches. No cytoplasmic protein contamination was detected, confirmed by the absence of the cytoskeleton marker β-actin, within the exosomal protein extracts when compared to total cell lysates (Fig. 1g). Interestingly, we also showed that fluorescently-labelled exosomes, added to culture media, can be internalised by surrounding fibroblasts, a process that can be blocked by anti-annexin antibodies^29^ (Supplementary Fig. 3). Altogether, these results demonstrated that dermal fibroblasts are able to release exosomes within the culture media, which can be uptake and internalised by surrounding cells to deliver their cargo.

### Upregulation of genes associated with ECM assembly, organisation and remodelling

**Figure 2:**
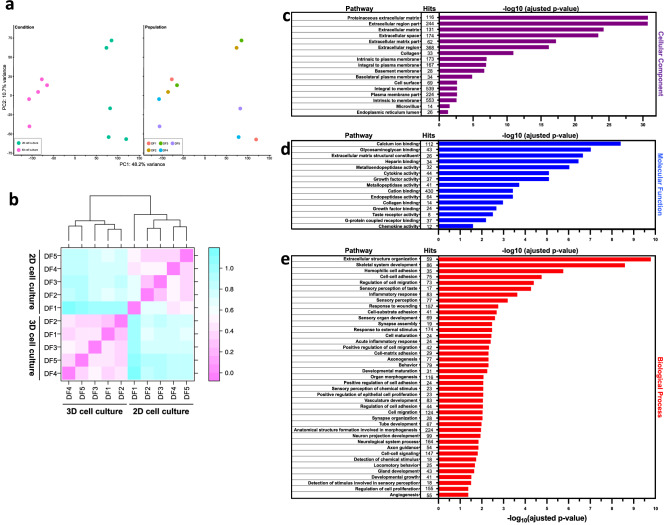
Differential gene expression according to the tested cell culture approaches and gene ontology enrichment analyses. Principal component analysis biplot showing a distinct gene expression clustering for each cellular culture condition (**a**). *N* = 5. DF = dermal fibroblast. Sample distance matrix showing the levels of relatedness, measured by Pearson correlation, between the tested culture methods based upon all differentially expressed genes (**b**). Gene ontology enrichment analysis of differentially up-regulated gene in 3D dermal fibroblasts culture (**c**–**e**). GO cellular component (**c**), molecular function (**d**) and biological process (**e**) enrichment analyses were performed using Network Analyst. Significantly enriched GO terms are shown with Benjamini–Hochberg FDR-corrected p-values and the number of hits is also included for each pathway.

In order to evaluate the effect of cell culture approaches on gene expression, we looked at transcriptional profiles of dermal fibroblasts cultivated in 2D and in 3D using high throughput RNA-seq analysis. At first, principal component analysis (PCA) showed distinct gene expression clusters depending on the tested cell culture approach (Fig. 2a). Furthermore, gene expression measured for the 3D fibroblast culture condition was more tightly clustered, which is indicative of a greater homogeneity among gene transcripts. To assess overall similarity/dissimilarity between samples, we then performed unsupervised hierarchical clustering using gene expression data (Fig. 2b). The sample distance measurements confirmed that each replicate showed high similarity and further confirmed clear dissociation between fibroblast gene expression profiles when cultured in 2D or in 3D (Fig. 2b). In-depth RNA-seq analysis revealed that 3730 genes were differentially modulated (fold change > 2; adjusted p-value < 0.05), out of which 2469 were upregulated in fibroblasts cultured in 3D and 1261 were downregulated. The 100 most upregulated and downregulated genes in 3D cultured fibroblasts are respectively listed in the Supplementary Tables 1 and 2. Next, we performed gene ontology (GO) enrichment analysis with the significantly modulated genes to gain additional insights into the biological processes, molecular functions and cellular components that are implied in the organisation of a 3D biological scaffold (Fig. 2c–e). Interestingly, we found that the upregulated genes in 3D cultures were exceedingly associated with GO cellular components linked to extracellular matrix protein and region, and the plasma membrane (Fig. 2c).

GO molecular functions were associated with numerous binding molecules (calcium ion, glycosaminoglycan, heparin, cation, collagen and growth factors), extracellular matrix structural constituent and metalloendopeptidase, cytokine, growth factor and metallopeptidase activities (Fig. 2d). Interestingly, GO biological processes were related to extracellular structure organisation, regulation of cell migration, inflammatory response, response to wounding and vasculature development (Fig. 2e). Overall, our findings revealed that the transcriptional profile of dermal fibroblasts grown in a 3D environment promoted the expression of a plethora of genes involved in the formation of the ECM.

### Exosomal protein content promotes the regulation of matrix remodelling, cell migration and angiogenesis

**Figure 3:**
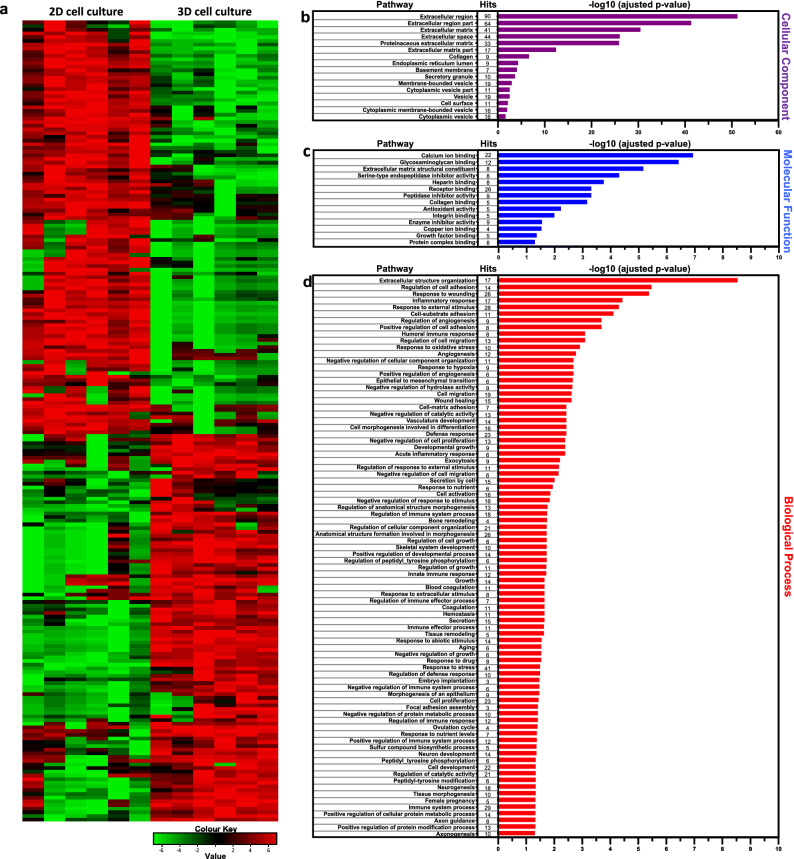
Proteomic and Gene Ontology enrichment analysis of exosomal protein isolated from dermal fibroblasts. (**a**) Heat map showing significantly deregulated proteins depending on the culture method. (**b**–**d**) Gene ontology enrichment analysis of differentially overexpressed exosomal proteins isolated from conditioned media of 3D dermal fibroblasts cultures. GO cellular component (**b**), molecular function (**c**) and biological process (**d**) enrichment analyses were performed using Network Analyst. Significantly enriched GO terms are shown with Benjamini–Hochberg FDR-corrected p-values and the number of hits is also included for each pathway.

A comprehensive proteome analysis to identify and quantify exosomal proteins collected in cell culture-conditioned media was performed. Likewise observed for gene expression profiles, PCA clearly revealed two separate exosomal protein clusters according to the tested cell culture approaches (Supplementary Fig. 4). Indeed, distinct exosomal protein expression profiles were found when comparing the two culture approaches, as shown in the displayed heat map (Fig. 3a). Overall, 853 proteins were identified in both 2D and 3D conditioned-media, out of which 442 were concentrated enough to be quantified. In-depth mass spectrometry analysis of 3D-derived exosomal protein revealed that 217 proteins were significantly modulated, out of which 105 were upregulated (Supplementary Table 3) and 112 downregulated (Supplementary Table 4). Subsequently, a detailed GO enrichment analysis was performed using data extracted from 3D-derived exosomal upregulated proteins (Fig. 3b–d). Of particular interest, GO cellular components correlated to extracellular region, ECM, matrix proteins, secretory granules, vesicles and cell surface (Fig. 3b), while GO molecular functions were associated with ECM structural constituent and binding activity (Fig. 3c). Lastly, the more significant GO biological processes were related to extracellular structure organisation, regulation of cell adhesion, response to wounding, inflammatory response, cell migration, angiogenesis, cell–matrix adhesion, exocytosis and secretion by cell (Fig. 3d).

**Figure 4:**
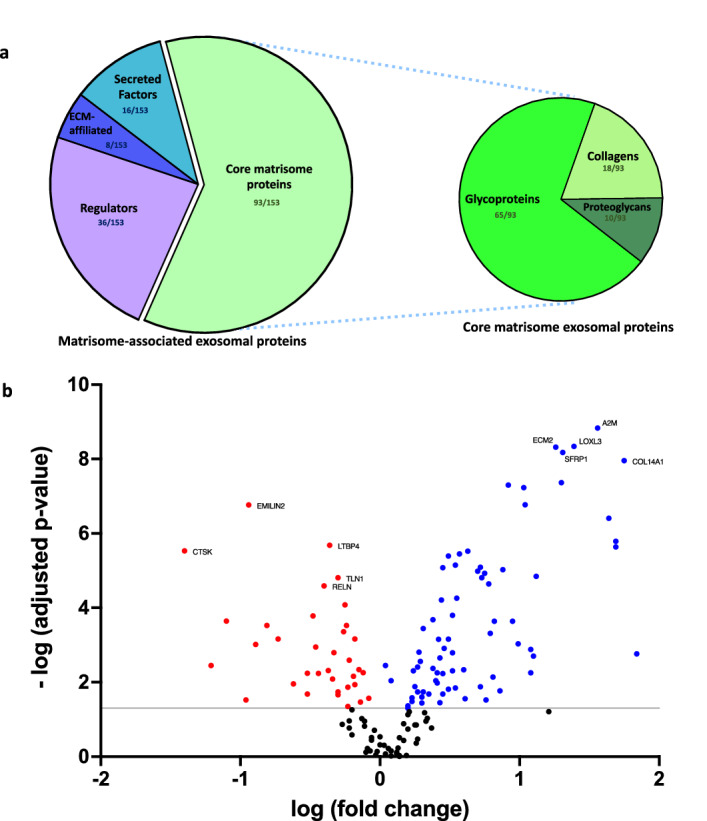
Exosomes secreted by skin fibroblasts cultured in 3D transports more proteins that are parts of the matrisome. Pie charts showing the number of matrisome-associated and core matrisome proteins that were quantified within the exosomes derived from fibroblasts (**a**). Volcano plot of matrisome-associated and core matrisome exosomal proteins (**b**). The blue dots represent significantly upregulated proteins, the red dots represent significantly downregulated proteins, and the black dots represent insignificant differentially expressed proteins. The grey line indicates an adjusted p-value of 0.05. The 5 most significantly upregulated and downregulated proteins are denoted.

To explore how the 3D cell culture-derived exosomes could influence the formation of the ECM, we used Ingenuity Pathway Analysis (IPA) curated database and our filtered dataset to build interactomes and predict multiple functions associated with ECM (Fig. 4 and Supplementary Fig. 5). On the one hand, formation, attachment, accumulation, remodelling and quantity of ECM are functions that are predicted to be enhanced in silico (Fig. 4). On the other hand, synthesis, deposition, adhesion, development, developmental process, organisation, cleavage, degradation, disassembly and mineralization of ECM are predicted to be inhibited (Supplementary Fig. 5). Interestingly, the vast majority of connections between proteins and functions are pointing towards the cytokine IL-6 (Fig. 4 and Supplementary Fig. 5), which is 5.19 times (log_2_ = 1.71) more present in 3D-exosomes (Supplementary Table 3). To further confirmed the pro-angiogenic property of 3D-derived exosomes, which was first highlighted during our GO terms analysis, biologically relevant tube formation assays demonstrated that exosomes derived from 3D cultures enhanced angiogenesis in vitro (Supplementary Fig. 6). In brief, these results put emphasis on the key role of exosomes and exosomal proteins in regulating pathways involved in ECM formation, assembly and maintenance, where IL-6 could actively participate in the process, as well as angiogenesis.

### Unbiased analysis of overall dermal matrisome detected in 3D-derived exosomes

**Figure 5:**
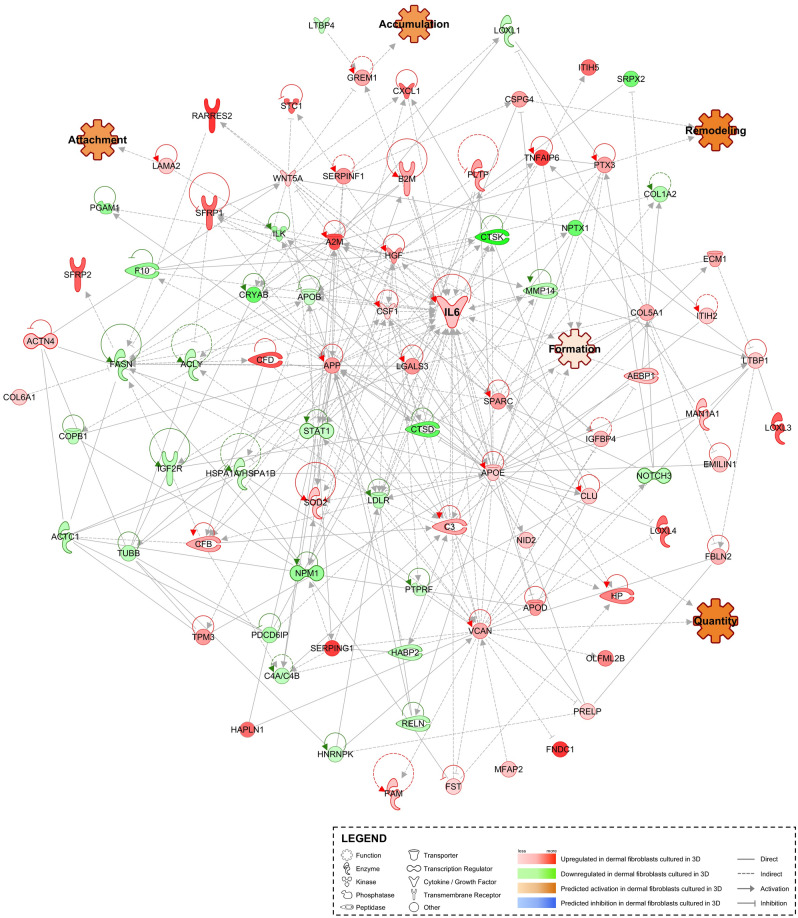
Interactome analysis of exosomal proteins isolated from exosomes secreted within 3D fibroblast culture media. Ingenuity Pathway Analysis generated interactome of significantly modulated exosomal proteins detected from 3D fibroblast cultures. Protein expression profiles predict the activation of functions associated with extracellular matrix, such as quantity, formation, attachment, accumulation and remodelling. The cytokine IL-6, which seems to be central to the signalling pathways, has been highlighted.

To further evaluate the role of 3D-exosomes in matrix remodelling, proteins that were quantified by mass spectrometry were then analysed using the MatrisomeDB 2.0 database (Hynes and Naba, 2012; Naba et al. 2016). Overall, 153 proteins were associated with the matrisome, from which 93 were parts of the ‘’core matrisome’’ (Fig. 5a). Dermal fibroblasts-derived exosomes have 16 secreted factors, 8 ECM-affiliated proteins, 36 regulators, 65 glycoproteins, 18 collagens and 10 proteoglycans. Amongst these 153 proteins, 109 matrisome-related proteins were significantly modulated in exosomes isolated from 3D cultures, from which 74 were up-regulated and 35 were down-regulated (Fig. 5b). Significantly regulated matrisome-associated proteins are listed in Supplementary Tables 3 and 4. Interestingly, the modulation of those proteins at the transcriptional level were very different (Supplementary Fig. 7). In fact, 80 matrisome-associated genes were up-regulated in 3D cell cultures and only 10 genes were significantly decreased. Together, the results from the above analyses suggest that preservation of soluble matrisome-associated components, detected in 3D-derived exosomes, which could actively participate to the various processes linked with the ECM and support skin-specific functions.

### 3D-derived exosomal MMPs regulate matrix remodelling

**Figure 6:**
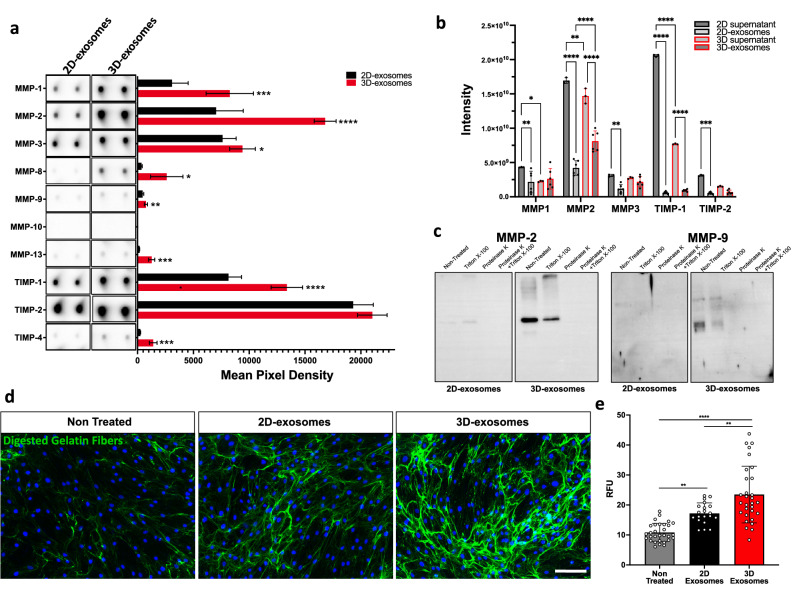
MMP expression profiles and enzymatic activity detected in dermal fibroblast-conditioned media. Quantification of exosomal MMPs and TIMPs using human MMP proteome profile arrays according to the culture method (**a**). An equal amount of exosomal proteins were used for each membrane. *n* = 3. Images of all membranes are shown in the Supplementary Fig. 8. (**b**) MMPs and TIMPs expression in exosomes and supernatant measured by MS for both cell cultures approaches (**c**)**.** Exosomal localization of MMP-2 and MMP-9. Equivalent amounts of all samples were resolved with SDS-PAGE and subjected to Western blotting with anti-MMP2 and anti-MMP9 antibodies. Note that the cropped images only are shown for conciseness; see supplementary figures section to see the original blots **(d)**. In situ gelatine zymography assay of fibroblasts treated with 2D cell culture- and 3D cell culture-derived exosomes. Nuclei were counterstained with DAPI (blue). *n* = 20–30. Scale bar = 50 μm. (**e**) Digested/remodelled gelatine fibers released a quencher which emits fluorescence (green). Positive fluorescence was reported as the ratio of the total area (RFU) Statistical analyses were performed by a one-way ANOVA with Tukey’s multiple comparison test. Graphs show mean ± SD. * *P* < 0.05, ** *P* < 0.01 and **** *P* < 0.0001.

To further confirmed the role of exosome into matrix remodelling, we investigated the MMPs and TIMPs protein content in exosomes derived from 3D cultured dermal fibroblasts by performing a proteome MMPs/TIMPs antibody array. MMP-1, MMP-2, MMP-3, MMP-8, MMP-9 and MMP-13 expression levels were consistently found to be significantly increased in exosomes derived from dermal fibroblast grown in a 3D state (Fig. 6a and Supplementary Fig. 8). TIMP-1 and TIMP-4 expression levels were also increased in 3D-exosomes (Fig. 6a and Supplementary Fig. 8). These results were further validated by RNAseq analysis (Supplementary Fig. 9). We then crossed-referred these antibody array data with the mass spectrometry datasets focusing on exosomal and soluble MMPs and TIMPs detected in the different conditioned media. Only MMP-1, MMP-2, MMP-3, TIMP-1 and TIMP-2 were detected by nanoLC-MS/MS in the conditioned media and within exosomes (Fig. 6b). Overall, the gelatinase MMP-2 was found to be the most expressed MMPs detected in 3D-exosomes when compared to 2D-exosomes (*p* < 0.0001) (Fig. 6a,b). Lower soluble MMP2 was detected in 3D-conditioned media when compared to 2D-conditioned media. In contrast, a higher exosomal MMP2 expression is detected in 3D-derived exosome, indicative of an exosomes secretion-mediated pathway. Proteinase K treatment has demonstrated that the gelatinases MMP-2 and MMP-9 are located at the external surface of the exosome’s membranes (Fig. 6c).

To also measure exosomal MMP-gelatinases enzymatic activity, including both MMP-2 (gelatinase A) and MMP-9 (gelatinase B), in situ gelatine zymography was performed. In situ gelatine zymography analysis revealed a significantly improved matrix-degrading MMP proteolytic activity for both cell culture approaches (2D-exosomes: RFU of 17.20 (*p* < 0.01); 3D-exosomes: RFU of 23.48 (*p* < 0.0001)) in comparison to the non-treated control (RFU of 10.80) (Fig. 6d,e). These results also revealed that proteolytic activity measured in 3D-exosomes was significantly higher than 2D-exosomes (*p* < 0.01). Collectively, our data revealed that the tested cell culture approaches considerably affected the releasing of MMPs via exosomes, which in turn may further impact matrix remodelling as previously predicted in the in silico analysis presented in the above section.

### Exosomal IL-6 enhances cellular migration and proliferation

**Figure 7:**
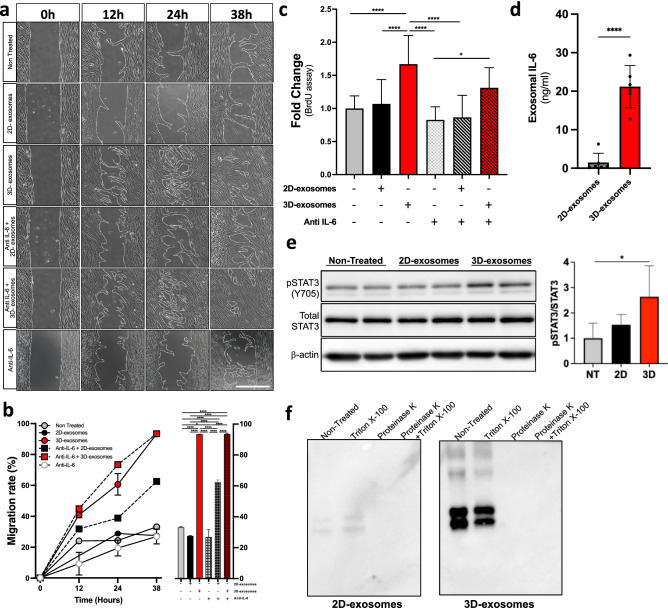
Cellular migration and proliferation are partially regulated through the IL-6 signalling pathway. In vitro scratch assay evaluating the effect of exosomes and anti-IL-6 treatments on the migration of dermal fibroblasts according to the culture method (**a**,**b**). Fibroblast were pre-treated with 250 μg of exosomes with or without 2 μg/mL anti-IL-6 prior to the assay and retreated with 250 μg of exosomes at the initial time point. Exosomal treatments were composed of a pool of an equal part of the six populations. Images were captured at various time points (0, 12 h, 24 h and 38 h) and closing rate was measured by reporting the total scratch area at each time point to the area at the initial time. Scale bar = 500 μm. *n* = 3. Cell proliferation ratio of fibroblasts treated with 2D- or 3D-exosomes and anti-IL-6 using a BrdU cell proliferation ELISA assay (**c**). Ratios were reported as a fold change compared to the non-treated condition. *n* = 9–18. Quantification of secreted IL-6 by ELISA (**d**). Exosomal IL-6 concentration is represented in ng/mL and normalised to the number of cells (gDNA). *n* = 6. Phosphorylation level of STAT (Tyr705) in fibroblasts treated with both types of exosomes (**e**). Phosphorylation levels were report to total STAT3 and are shown as fold change, relative to the non-treated control. *n* = 3. Exosomal localization of IL-6. Equivalent amounts of all samples were resolved with SDS-PAGE and subjected to Western blotting. Note that the cropped images only are shown for conciseness; see supplementary figures section to see the original blots **(f).** Microparticles were treated with 100 mg/mL proteinase K and/or 1% Triton X-100. An equal amount of total exosomal proteins were then analysed by Western blot. Note that the cropped images only are shown for conciseness; see supplementary figures section to see the original blots Note again that the cropped images only are shown for conciseness; see supplementary figures section to see the original blots. Statistical analyses were performed by a one-way ANOVA with Tukey’s multiple comparison test. Graphs show mean ± SD. * *P* < 0.05, ** *P* < 0.01 and **** *P* < 0.0001.

Next, we sought to evaluate the migratory and proliferative properties of fibroblast-derived exosomes. Scratch assays revealed that 3D-exosomes highly promoted cellular migration, in comparison to 2D-exosomes and to the non-treated control (*p* < 0.0001) (Fig. 7a,b). In the same way, exosomes isolated from 3D cell cultures significantly enhanced cellular proliferation by 1.47-fold (*p* < 0.05) as measured by BrdU assay (Fig. 7c). To further evaluate the proliferative properties of 3D-exosomes, monolayered fibroblasts were treated with *N*-arachidonyldopamine (NADA), an anti-proliferative agent. While pre-treatment with NADA considerably suppressed the scratch closure, the addition of 3D-exosomes within the culture media also containing NADA surprisingly restored the closure of the scratch, whereas the addition of 2D-exosomes had not effect (*p* < 0.0001) (Supplementary Fig. 10a,b). Similarly, treating monolayered cell culture with NADA considerably reduced proliferation (*p* < 0.0001), which was restored by the addition of 3D-exosomes in the culture media (*p* < 0.05) (Supplementary Fig. 10c).

To further study exosomal cytokines known to stimulate cellular migration and proliferation, we performed a cytokine array using exosomal proteins extracted from 2D- and 3D-exosomes. Interestingly and consistent with the in silico prediction, significant higher concentration (~ 20-fold, *p* < 0.0001) of the multifunctional cytokine interleukin-6 (IL-6) in exosomes derived from 3D cultures was detected (2D: 1.497 ± 2.379 vs 3D: 21.200 ± 5.468 ng/mL) (Fig. 7d and Supplementary Fig. 11). Higher concentrations of the growth-regulated protein alpha (GRO-α), the interleukin-8 (IL-8) and the monocyte chemoattractant protein-1 (MCP-1) were also measured in 3D-exosomes (Supplementary Fig. 11). Interestingly, phosphorylation level of STAT (Tyr705), enhanced by IL-6, in fibroblasts treated with 3D-exosomes was significantly increased (*p* < 0.05) (Fig. 7e) along with the expression of collagen alpha 1 (COL1A1) known to be stimulated by IL-6 (Supplementary Fig. 12). Upon proteinase K treatment, IL-6 was actually expressed at the surface of the exosomes (Fig. 7f).

Moreover, treating monolayered cell culture with anti-IL-6 antibodies suppressed the scratch closure and the addition of 3D-exosomes within the culture media enhanced cellular migration rate (*p* < 0.0001) (Fig. 7a,b). Likewise, exosomes isolated from 3D cell culture-conditioned media have partly re-established the proliferation rate that has been initially repressed using anti-IL-6 antibodies (*p* < 0.05) (Fig. 7c). Taken together, our results revealed that exosomal IL-6 partially stimulated cellular migration and proliferation, which are key biological functions associated with ECM formation.

## Discussion

In this study, we showed that 3D cellular culture drastically affected the content of dermal fibroblast-derived exosomes and demonstrated that ECM proteins are secreted into the intercellular space via exosome-mediated pathway. We also showed that exosomal ECM proteins are predicted to actively participate to ECM assembly, remodelling and stability, as well as to promote cellular migration and proliferation, partly through IL-6 signalling. We used quantitative proteomic profiles to reveal an in-depth extracellular matrix proteome (matrisome) associated with exosomes, secreted by 3D cultured dermal fibroblasts. We also showed, through in vitro biological studies, that 3D-derived exosomes held modulatory properties and regulatory effects on cell migration, cell proliferation, ECM secretion and remodelling as well as on angiogenesis.

Over the last few years, 3D cell culture systems have become widely used over standard 2D monolayer culture to better mimic tissues and complex diseases in vitro^30^. Although 2D monolayer cell cultures are more simple and still useful for investigating basic principles of cell biology, there are numerous inherent limitations associated with it, such as the lack of interactions between the cellular and extracellular environment, changes in cell morphology and polarity, and dedifferentiation^32^. The loss of disease-associated phenotypes often observed while culturing primary patient-derived monolayered cells, is also an important limitation of 2D cellular cultivation^31–34^. Cell-to-cell and cell-to-extracellular environment interactions have been shown to be responsible for cell differentiation, proliferation, survival, expression of genes and proteins, responsiveness to stimuli and other important cellular functions^37–40^. To overcome those limitations, 3D cell culture has emerged as an alternative strategy to better mimic cellular interactions with microenvironment and the ECM, and to recapitulate native biological and cellular functions^35–39^.

Exosomes are a subtype of EVs and exert important biological functions in health and diseases by mediating horizontal intercellular communication. Indeed, exosomes have been reported to play a major role in many cellular processes, such as immune and inflammatory responses^40,41^, signal transduction^42^, antigen presentation^43^ and wound healing^44,45^. EVs were shown to be structural and functional components of the ECM and some attention has been paid to the ability to regulate the target cells’ ECM synthesis and degradation^16,46^. However, it remains to be determined whether exosomes actively participate in signalling events driving ECM deposition, assembly and remodelling. Our data settled down the role of exosomes in these processes as well as determining how fibroblasts-derived exosomes could contribute to the formation of dermal ECM.

Combined transcriptome and proteome in-depth analyses revealed major changes in gene and protein expression profiles depending on the cell culture method—classical 2D monolayer cell culture vs scaffold-free tissue-engineered 3D cell culture. The most significant modulated genes, detected in 3D-derived exosomes, were associated to ECM deposition, accumulation, organisation and remodelling pathways, and highlighted the cytokine IL-6 and MMPs as key regulators of these processes. On the same note, exosomal protein expression associated to the production and secretion of ECM protein secretion, processing and assembly, were also highly modulated. Indeed, MS analysis showed that most of the proteins detected in 3D-derived exosomes were associated with cell-to-cell, cell-to-matrix and ECM interactions. In fact, proteins associated with the ECM were found to be more significantly abundant in 3D-derived exosomes, providing further evidence towards an exosome-dependent mechanism of ECM secretion and remodelling. Dermal fibroblasts are the main effector cells in cutaneous wound healing of soft tissue^47^. Fibroblast migration, proliferation and collagen synthesis are important for the quality of wound healing. We showed here that human exosomes, collected from conditioned-media following 3D dermal fibroblast culture, can be internalised by fibroblasts, and exert a paracrine role in promoting ECM synthesis and secretion from dermal fibroblast, as well as cellular migration and proliferation partly through IL-6 signalling.

IL-6 and the IL-6/JAK/STAT3 signalling pathway have a key role in the growth and development of many human cancers, and have been shown to drive cellular proliferation, survival, invasiveness, and metastasis of tumour cells^48^. Evidence is accumulating that IL-6 may also be involved in the pathogenesis of several types of skin disorders, such as fibrosis^49,50^, scleroderma^51^, psoriasis^52,53^ and systemic lupus erythematosus^54,55^. Increased IL-6 messenger RNA and protein expression were detected in fibroblasts surrounding the wound sites after skin incisions^56^, indicating that IL-6 could contribute to the function and growth of fibroblasts^57^. Interestingly and in line with our findings, IL-6 was shown to be predominantly located at the exosomes surface^58^. The release of cytokines, IL-6 for instance, at the surface of EVs should be more efficient than depositing free molecules into the extracellular space^58^. Numerous other lines of evidence demonstrated that IL-6 can induce collagen production and/or pro-collagen gene expression in several types of cells including dermal fibroblasts^57,59^ and be central to cutaneous wound healing.

There are also several lines of evidence that exosomes can promote ECM remodelling in cutaneous wound repair^60–62^. Interestingly, although exosomal MMP levels were found to be decreased in 3D-derived exosomes, increased MMP proteolytic activity were detected by gelatinase in situ zymography, which is indicative of increased ECM remodelling, a biological function predicted to be activated in our gene and protein expression datasets. Hence, our data showed that gelatinases (MMP-2 and MMP-9) were more active and were located within the external surface of the exosomes isolated from 3D culture of dermal fibroblasts. Accumulating evidence show that exosomes-associated metalloproteinases are involve in matrix remodelling^15,63^. Interestingly, increased MMP-2/9 activity was previously shown to promote in turn migration and proliferation of these cell populations^64,65^. Moreover, these MMPs are known to be involved in wound healing, mostly by playing a crucial role in cellular migration of skin keratinocytes^66^. These results further indicate that 3D-derived exosomes, produced by dermal fibroblasts, could also actively participate to ECM remodelling and cellular migration during wound healing.

Enhanced angiogenesis is another important change observed in the proliferative phase of the wound-healing process^67–69^. Pro-angiogenic proteins and growth factors were found significantly augmented in 3D-exosomes (Fig. 3 and Supplementary Table 3), pointing out towards another role mediated by 3D dermal fibroblast-derived exosomes. In particular, MMP-2 and MMP-9 are known to be involved in angiogenesis, often studied in the context of tumour propagation and during wound healing^70–72^, where the balance between MMP-2 and TIMP-2 seems to be imperative^71^. IL-6 is also involved in the formation of vasculature and a key factor for tumour growth^73,74^. It would have been interesting to use dermal fibroblasts silenced for MMP2, MMP9 or IL-6 to further confirm the presented results.

By culturing and differentiating in 3D in vitro biomimetic microenvironments, human dermal fibroblasts can secrete exosomes, which can actively promote ECM synthesis, cellular migration and proliferation by surrounding fibroblasts, thereby accelerating wound healing of soft tissue. Our findings suggest that exosomes contained within fibroblast-conditioned media cultivated in 3D may represent a novel, easily accessible research and therapeutic tool to study ECM secretion and cutaneous wound healing.

## Materials and methods

### Cell isolation and culture

All recruited individuals signed an informed consent form approved by our Institutional Ethics Committees prior to being enrolled in the study on a voluntary basis (Ethical research board of the CHU de Québec. Protocol number: 2012–1316. For more information, please contact (gurecherche@chuq.qc.ca). All experiments were performed in compliance with the national Tri-Council Policy Guidelines: Ethical Conduct for Research Involving Humans and approved by the ethics committee of the CHU de Quebec – Université Laval. Fibroblasts were isolated from skin biopsies of 6 informed consent healthy individuals (Supplementary Table 5) as previously described^33^. Fibroblasts were grown in Dulbecco-Vogt modification of Eagle’s medium (DMEM; Invitrogen, Burlington, ON, Canada) supplemented with 10% foetal bovine serum (FBS; VWR, Radnor, PA, USA), 100 IU/mL penicillin G (Sigma-Aldrich, Oakville, QC, Canada), and 25 μg/mL gentamicin (Schering, Pointe-Claire, QC, Canada). Cell cultures were kept in an incubator at 37 °C, 8% CO_2_ and 95% relative humidity, and media was changed every 2 days.

### Cell culture

Dermal fibroblasts were seeded at a concentration of 3 × 10^4^ cells/cm^2^ in 6-well plates in complete DMEM. For 2D cell culture, fibroblasts were kept in monolayer for a week, which also include exosome production. In parallel, for 3D cell culture, the media was supplemented with 50 μg/mL ascorbic acid (Sigma-Aldrich) and cells were cultured for 28 days, in order to produce a manipulable fibroblast sheet composed of a neosynthesised ECM proteins meshwork^32,33^. Paper anchors and plastic weight were used to keep the cell sheets from contracting, as described previously^32,33^. Dermal fibroblasts were kept in an incubator at 37 °C, 8% CO_2_ and 95% relative humidity, and media was changed every 2 days.

### Exosome isolation

Exosome isolation was done following already published methods, with few modifications^75^. Serum was depleted from bovine exosomes with the FBS Exosome Depletion Kit (Norgen Biotek Corp., ON, Canada) according to manufacturer’s instructions. Cells, either grown in 2D or in 3D, were then incubated with complete DMEM without phenol red (Wisent Bioproducts, Montréal, Québec, Canada) supplemented with 5% of bovine exosome-depleted FBS and antibiotics, in 8% CO2 at 37 °C for 72 h. Fibroblast-conditioned media were centrifuged at 2000×*g* for 20 min at 4 °C to remove cell debris, before being treated overnight at 4 °C with the Total Exosome Isolation Reagent from cell culture media (Invitrogen) according to manufacturer’s instructions. Samples then were centrifuged at 10,000×*g* for 1 h at 4 °C, exosomes pellets were recovered in sterile PBS and kept at − 80 °C until use.

### Nanoparticle tracking analysis

Exosome size distribution and concentration were investigated on the NanoSight NS300 Instrument according to the manufacturer’s protocol (Malvern Instrument, Malvern, Worcestershire, UK). Briefly, 3 to 5 videos of 30 s were taken for each sample diluted in PBS. A pool of 6 populations of fibroblasts-derived exosomes was analysed for each culture method. Capture parameters were set at a rate of 30 frames per seconds and particle movement was analysed with the NTA software (v 2.3; NanoSight Ltd, Amesbury, Wiltshire, UK). Nanoparticle size distribution curve, refractive index and the relative nanoparticle concentration of a particular size were recorded, with the cumulative percentage of nanoparticles.

### Acetylcholinesterase exosomal activity

Fibroblasts grown in 3D were incubated for 48 h with complete DMEM supplemented with 10% of exosome-depleted FBS, antibiotic cocktail and 30 μM GW4869 (Sigma-Aldrich). Secreted exosomes were isolated from the conditioned culture media as mentioned above and then the acetylcholinesterase (AChE) exosomal activity was measured. In brief, 50 μg of exosomes were resuspended in PBS, 5 μL was distributed in triplicate in a 96-well plate containing 1.25 mM S-acethylthiocholine iodide (Alfa Aesar, Haverhill, MA, United States) and 0.1 mM 5,5’-dithiobis-(2-nitrobenzoic acid) (DTNB; BioBasic Inc., Markham, Ontario, Canada). Absorbance was read at 412 nm every 5 min for 45 min at 37 °C.

### Effect of GW4869 treatment on exosomes secretion

Fibroblasts were seeded at a density of 3 × 10^4^ cells/cm^2^ in 12-well plates in complete DMEM. After 48 h, cells were pre-treated with 30 μM GW4869 (Sigma-Aldrich) or DMSO for 48 h. For exosomes production, fibroblasts were then incubated with complete DMEM without phenol red (Wisent Bioproducts) supplemented with 5% of bovine exosome-depleted FBS, antibiotics and 30 μM GW4869 (Sigma-Aldrich) or DMSO, in 8% CO2 at 37 °C for 72 h. Exosomes were isolated with Total Exosome Isolation Reagent from cell culture media (Invitrogen) as described above. Finally, total exosomal proteins was quantified using a standard Bradford assay (Bio-Rad).

### Transmission electron microscopy

Exosomes were first fixed in 2.5% glutaraldehyde overnight at 4 °C. Samples were centrifuged at 21,000×*g* for 20 min at 4 °C and the pellet was washed 6 times with a 0.1 M cacodylate buffer. Exosomes were then loaded onto a copper grid covered with a carbon film and stained with 3% uranyl acetate. Electron micrographs were assessed using a JEOL (JEM-1230; Tokyo, Japan) transmission electron microscope at an excitation of 80 kV.

### Next-generation RNA sequencing and gene expression profiling

RNA of 2D and 3D fibroblast cultures was extracted using TRIzol reagent (Invitrogen) and total RNA was then isolated using the RNA Miniprep Kit (BioBasic Inc.) following manufacturer’s instructions. RNA quality was assessed on a Bioanalyzer using the Agilent RNA 600 Nano Kit (Agilent) and the concentration and purity was determined using a NanoDrop 2000 (Thermo Fisher Scientific, Waltham, MA). Ribosomal RNAs were depleted from total RNA using Ribo-Zero rRNA removal kit specific for HMR RNA (Illumina, San Diego, CA, USA) and residual RNA was cleaned up using the Agencourt RNACleanTM XP Kit (Beckman Coulter, Brea, CA, USA) and eluted in water. Libraries were generated using the KAPA Stranded RNA-Seq Library Preparation Kit (Kapa Biosystems, Wilmington, MA, USA), as detailed in the manufacturer’s recommendations. TruSeq adapters and PCR primers were purchased from Integrated DNA Technologies, Inc. (IDT, Coralville, IA, USA). Libraries were quantified using the Quant-iT™ PicoGreen® dsDNA Assay Kit (Life Technologies, Carlsbad, CA, USA) and the Kapa Illumina GA with Revised Primers-SYBR Fast Universal kit (Kapa Biosystems). Average size fragment was determined using a LabChip GX instrument (PerkinElmer, Waltham, MA, USA).

Bioinformatic investigations were done on the free online platform namely Network Analyst (https://www.networkanalyst.ca/). Raw data were imported, and the low abundance (5th percentile) and low variance genes (15th percentile) were filtered out. Then, the data were subjected to an upper quantile normalisation and a pairwise differential expression analysis with the DESeq2 statistical method. Genes with a fold change of 2 and more (log_2_ = 1) and an adjusted p-value inferior or equal to 0.05 were considered significantly modulated. Finally, a gene ontology (GO) enrichment analysis was performed.

### Mass spectrometry sample processing and data analysis

Exosomes were lysed using a buffer containing 50 mM ammonium bicarbonate, 0.5% sodium deoxycholate, 50 mM dithiothreitol, 1 μM pepstatin (Sigma-Aldrich) and 1X protease inhibitor cocktail tablets (cOmplet, Mini, EDTA-free, Roche, Basel, Switzerland), and then vortexed for 1 min and sonicated with the following settings: 20% amplitude, 20 cycles of 1 s on/off. Debris were removed following a centrifugation of 15 min at 13,000 rpm at 4 °C. Samples were analysed using a nanoscale capillary liquid chromatography (nanoLC-MS/MS). For the six populations of exosomes, 1 µg of peptide samples was injected and separated by online reversed-phase nanoLC and analysed by electrospray mass spectrometry (ESI MS/MS). The experiments were performed with a Dionex UltiMate 3000 nanoRSLC chromatography system (Thermo Fisher Scientific / Dionex Softron GmbH, Germering, Germany) connected to an Orbitrap Fusion mass spectrometer (Thermo Fisher Scientific) driving with Orbitrap Fusion Tune Application 3.3.2782.34 and equipped with a nanoelectrospray ion source. The detailed nanoLC-MS/MS acquisition parameters are listed in the Supplementary methods section.

Raw data were first analysed in the Andromeda module of MaxQuant software (v. 1.5.3.30). Trypsin/P enzyme parameter was selected with two possible missed cleavages. Carbamidomethylation of cysteins, methionine oxidation and acetylation of protein N-terminus were set as variable modifications. Mass search tolerances were of 5 ppm and 0.6 Da for MS and MS/MS respectively. For protein validation, a maximum False Discovery Rate of 1% at peptide and protein level was used based on a target/decoy search. MaxQuant was also used for label-free quantification. The ‘match between runs’ option was used with a 20 min value as alignment time window and 3 min as match time window. Only unique and razor peptides were used for quantification.

Bioinformatic analysis was initially performed on Network Analyst (https://www.networkanalyst.ca/). The quantified proteins dataset was filtered out for the low abundance (5th percentile) and low variance protein (15th percentile) and was subjected to a pairwise differential expression analysis with a limma statistical analysis. Proteins with a fold change of 2 and more (log_2_ = 1) and an adjusted p-value lower or equal to 0.05 were considered significant. A gene ontology (GO) enrichment analysis was also achieved. Results from the network analysis were then uploaded into the Ingenuity Pathway Analysis (IPA) (QIAGEN Inc., https://www.qiagenbioinformatics.com/products/ingenuitypathway-analysis) software to produce interactomes with significantly modulated exosomal proteins that were associated with all the functions involving ECM. The in silico prediction abilities of IPA were used to assess how these functions would be affected by the method of culture. The dataset was also analysed using the MatrisomeDB 2.0 website (http://matrisomeproject.mit.edu/proteins/).

### Multiplexed MMP and TIMP expression array

Exosomal MMP and TIMP levels were measured using the Human MMP Antibody Array (Abcam), targetting 10 proteins, according to the manufacturer’s instructions. Exosome samples, previously isolated from 1 mL of conditioned media, were first lysed with RIPA 1X (Abcam) and resuspended in a total of 1 mL of PBS. Briefly, membranes were first blocked using the given Blocking Buffer and then incubated with the resuspended samples overnight at 4 °C. The following day, membranes were washed and incubated with the biotin-conjugated antibodies for 2 h at room temperature and then incubated in HRP-Conjugated Streptavidin overnight at 4 °C. Chemiluminescence was imaged using Fusion Fx7 imager (Vilber Lourmat), protein dots were quantified using ImageJ spot recognition analysis.

### In situ gelatine zymography assay

Fibroblasts were seeded at a density of 1 × 10^3^ cells/well on Millicell EZ SLIDE 8-well glass slide culture (EMD Millipore) coated with 100 mg/mL of quenched fluorescein-labelled gelatine (DQ™ Gelatin D-1–12,054, Molecular Probes, Eugene, OR, USA). Slides were then incubated overnight with 500 μg of 2D- or 3D-exosomes. Cells were rinsed in PBS with 0.05% Tween-20 and mounted with Fluoromount-G containing DAPI (Invitrogen). Samples were observed using a Zeiss Axio Imager M2 microscope equipped with an Axiocam HR Rev3 camera (Oberkochen, Germany). Fluorescence peptides, released by the enzymatic cleavage of DQ™ gelatine, were quantified using ImageJ 1.46 software and normalised for the number of cells per well.

### Cell migration assay

Fibroblasts were seeded at a density of 7.5 × 10^4^ cells/well in 35 mm culture-insert μ-Dish (Ibidi, Martinsried, Planegg, Germany) in complete DMEM supplemented with 250 μg of 2D- or 3D-exosomes in presence or absence of 2 μg/mL of IL-6 antibodies (anti-IL-6) or 10 μM of the cell proliferation inhibitor NADA. After 18 h of incubation time, the culture-inserts were removed to create a 500 μm cell-free gap. Cells were treated for the second time with 250 μg exosomes and were allowed to migrate for an additional 38 h. Images were captured at different time points (0, 12, 24 and 38 h) using an inverted microscope (TE2000; Nikon, Minato City, Tokyo, Japan). Fibroblast migration rate was calculated by measuring the closing area and reported as a percentage of the initial scratched area using ImageJ 1.52v software.

### Cell proliferation assay

Fibroblasts were seeded at a density of 5 × 10^3^ cells/well in a 96-well plate containing complete DMEM supplemented with 5% exosome-depleted FBS and 500 μg/mL of 2D- or 3D-exosomes in presence or absence of 2 μg/mL of anti-IL-6 or 10 μM of NADA and then cells were incubated for 18 h. The cellular proliferation rate was then assayed using a BrdU Cell Proliferation ELISA kit (Abcam) following manufacturer’s instructions.

### ELISA against IL-6

Exosomal cytokines were quantified using a specific Human IL-6 ELISA kit (Abcam). All diluted samples were run in duplicate, and plates were read at a wavelength of 450 nm using a microplate reader (Bio-Rad). Results were normalised with the number of cells (gDNA) present in the culture dishes.

### Proteinase K treatment

Exosomal particles, derived from both cell culture approach, were first permeabilised, or not, with 1% Triton X-100 (Bio-Rad) and treated with 100 mg/mL Proteinase K (BioBasic Inc.) at 56 °C for 1 h. Proteinase K was heat inactivated for 10 min at 95 °C. All samples were then analysed by western blot.

### Exosomes and IL-6 treatment

Fibroblasts were seeded at a density of 3 × 10^4^ cells/cm^2^ in 6-well plates in complete DMEM. Five days later, cells were treated with 750 μg of both type of exosomes for 48 h. After the treatment a proportion of cells were harvested for western blot analysis and the rest were cultured for an additional 48 h to analyse collagen production.

### Western blot analysis

Total exosomal proteins were extracted in 1X RIPA buffer (Abcam) containing 1X protease inhibitor cocktail tablets (cOmplet, Mini, EDTA-free, Roche), and were quantified using the Pierce™ BCA protein assay kit (Thermo Fisher Scientific) according to the manufacturer’s instructions. Exosomal protein lysates (20 μg) were subjected to a 12% SDS-PAGE, transferred to a polyvinylidene fluoride membrane (PVDF, Bio-Rad Laboratories, Hercules, California, USA) or nitrocellulose membrane (Bio-Rad Laboratories) and then blocked with 5% non-fat dried milk and 0.05% Tween-20 in tris-buffered saline (TBS). Membranes were incubated overnight at 4 °C with primary antibodies against CD9 (1:1000, Biolegend), CD63 (1:1000, Biologend), CD81 (1:1000, Biolegend), MMP-2 (1:1000, Cell Signaling Technology), MMP-9 (1:1000, Cell Signaling Technology), IL-6 (1:1000, Cell Signaling Technology), phospho-STAT3 (1:1000, Cell Signaling Technology), STAT3 (1:1000, Cell Signaling Technology) and β-actin (1:2000, Cerderlane), followed by anti-rabbit IgG-HRP (1:5000; Jackson Immunoresearch Laboratories, West Grove, PA, USA) or anti-mouse IgG-HRP (1:5000; Jackson Immunoresearch Laboratories) secondary antibodies. Protein expression profile was detected using Amersham ECL Western Blotting Detection Reagent (GE Healthcare, Chicago, IL, USA) and imaged using Fusion Fx7 imager (Vilber Lourmat, France).

To quantify the expression of specific exosomal markers, 0.5% trichloroethanol (TCE) was added to the gels before polymerisation and fluorescence images of the migrated gels were taken with the Gel Doc EZ imager (Bio-Rad Laboratories).

### Statistical analysis

GraphPad Prism v.9.00 software and the open-source tool Network Analyst (http://www.networkanalyst.ca/) were used for statistical analysis. Data are represented as mean ± standard deviation (SD). Differences were considered statistically significant when p-value < 0.05 or adjusted p-value < 0.05. Is this study, ‘’N’’ is used when referring to different biological samples, and ‘’n’’ when referring to technical replicates.

## Supplementary Information

Supplementary Figures.

Supplementary Information 1.

Supplementary Table 1.

Supplementary Table 2.

Supplementary Table 3.

Supplementary Table 4.

Supplementary Table 5.
