# Supplementary material for: Tridimensional cell culture of dermal fibroblasts promotes exosome-mediated secretion of extracellular matrix proteins
Source: Sci Rep. 2022 Nov 17;12:19786. doi: 10.1038/s41598-022-23433-0 (PMC9672399; doi:10.1038/s41598-022-23433-0)
Supplement: Supplementary file 1 — Supplementary Figures. [file 41598_2022_23433_MOESM1_ESM.docx]

**Supplementary Figure 1**

**Expression of various genes involved in exosomes biogenesis in 2D and 3D fibroblast cultures**.

Linear signal of gene associated to different steps of exosomes biogenesis were retrieved from the RNAseq dataset and then normalised with the transcript levels of the housekeeping gene *B2M*. An unpaired T-test with Welch’s correction was performed for each gene and graph shows box and whiskers with max and min. *N* = 5. **P* < 0.05, ***P* < 0.01 and *****P* < 0.0001.

**Supplementary Figure 2**

**Quantification of specific exosomal markers.**

Western blot of exosomal CD9, CD63, CD81 and TCE staining for all populations and culture conditions. 12.5 μg of total exosomal protein was loaded in each well. M = molecular weight marker Equivalent amounts of all samples were resolved with SDS-PAGE and subjected to Western blotting with anti-CD9, anti-CD63 and anti-CD89 antibodies. Note that the cropped images only are shown for conciseness; the original blots are provided down below (**a**). Relative expression of exosomal markers, CD9 (**b**), CD63 (**c**) and CD81 (**d**), in 2D- and 3D-exosomes. TCE was used as a loading control for the normalisation and protein levels are shown as fold-change expression, relative to 2D-exosomes. An unpaired T-test with Welch’s correction was performed and data show mean ± SD. *N* = 6. **P < 0.01

**Supplementary Figure 3**

**Internalisation of exosomes by dermal fibroblasts**.

Fibroblasts were pre-treated with annexin A1, A2, V or VI antibodies to prevent exosomes internalization. Exosomes were pre-marked with PKH26 (red) and then fibroblasts were immunostained with vimentin (green). Nuclei were stained with DAPI (blue). Scale bar = 100 μm.

**Supplementary Figure 4**

**Principal component analysis biplot for exosomal proteins measured by nanoLC-MS/MS**.

PCA showing a distinct exosomal protein expression clustering for each cellular culture condition. *N* = 6.

**Supplementary Figure 5**

**Predicted inhibition of functions associated with ECM and related to exosomal proteins.**

IPA generated interactome of significantly modulated exosomal proteins detected from 3D fibroblast cultures. Protein expression profiles predict the inhibition of functions associated with ECM such as synthesis, deposition, adhesion, development, developmental process, organisation, cleavage, degradation, disassembly and mineralisation. The cytokine IL-6, which seems to be central to signalling pathways, has been highlighted.

**Supplementary Figure 6**

**3D-exosomes treatment enhances angiogenesis *in vitro*.**

Tube formation assay on Matrigel® of HMVECs seeded with exosomes produced by skin fibroblasts cultured in 3D and by GW4869-treated fibroblasts. Quantification of the area of tubes-like vessels was performed for each condition. The untreated condition refers as HMVECs not treated with exosomes. The same proportion in volume of exosomes isolated from fibroblasts treated with 30 μM GW4869 was used. Scale bar = 500 μm. (*N* = 3, *n* = 3). Statistical analyses were performed by a one-way ANOVA with Tukey’s multiple comparison test. Graphs show mean ± SD. * *P* < 0.05, and ** *P* < 0.01.

**Supplementary Figure 7**

**Volcano plot of matrisome-associated and core matrisome exosomal genes.**

The blue dots represent significantly upregulated genes, the red dots represent significantly downregulated genes, and the black dots represent insignificant differentially expressed genes. The grey line indicates an adjusted p-value of 0.05. The 5 most significantly upregulated and downregulated proteins indicated in the figure 5b are denoted.

**Supplementary Figure 8**

**Expression profiles of exosomal MMPs and TIMPs depending on culture conditions**

Images of all human MMP array membranes for exosomes derived from 2D and 3D dermal fibroblasts cultures. An egal amount of exosomal proteins were used for each membrane. Quantifications are shown in figure 6a.

**Supplementary Figure 9**

**Gene expression of MMPs and TIMPs for both cell culture approaches.**

Linear signal of MMPs and TIMPs genes were retrieved from the RNAseq dataset and then normalised with the transcript levels of the housekeeping gene *B2M*. An unpaired T-test with Welch’s correction was performed for each gene and graph shows box and whiskers with max and min. *N* = 5. **P* < 0.05 and ***P* < 0.01.

**Supplementary Figure 10**

**Exosomes derived from skin fibroblast cultured in 3D restore cellular migration and proliferation affected by NADA pre-treatments.**

*In vitro* scratch assay evaluating the effect of exosomes and NADA, an anti-proliferative agent, treatments on the migration of dermal fibroblasts according to the culture method (**a-b**). Fibroblast were pre-treated with 250 μg of exosomes with or without 10 μM NADA prior to the assay and retreated with 250 μg of exosomes at the initial time point. Exosomal treatments were composed of a pool of an egal part of the six populations. Images were captured at various time points (0, 12h, 24h and 38h) and closing rate was measured by reporting the total scratch area at each time point to the area at the initial time. Scale bar = 500 μm. *n* = 3. Cell proliferation ratio of fibroblasts treated with 2D- or 3D-exosomes and NADA using a BrdU cell proliferation ELISA assay. n = 9-18. (c). Ratios were reported as a fold change compared to the non-treated condition. Statistical analyses were performed by a one-way ANOVA with Tukey’s multiple comparison test. Graphs show mean ± SD. * P < 0.05, ** P < 0.01 and **** P <0.0001.

**Supplementary Figure 11**

**Exosomes derived from 3D cultures contained a high level of IL-6 and other cytokines.**

Quantification of exosomal cytokines with a human proteome profiler array (**a**). The duplicate spots corresponding to the most altered proteins are shown. An egal amount of exosomal proteins were used for each membrane. *N* = 3. Images of all membranes for exosomes derived from 2D and 3D dermal fibroblasts culture (**b**). An unpaired T-test with Welch’s correction was performed and data show mean ± SD. ****P* < 0.001 and *****P* < 0.0001.

**Supplementary Figure 12**

**Exosomes derived from 3D cultures promote pro-collagen I alpha 1 secretion.**

Quantification of secreted pro-collagen I alpha 1 by ELISA following exosomes treatments. Dermal fibroblasts were treated with both types of exosomes for 48 hours and media were conditioned for an additional 48 hours. *n* = 6-7. A one-way ANOVA was performed and data show mean ± SD. ***P* < 0.01.

**COMPLETE MEMBRANES FOR WESTERN BLOT ANALYSIS**

**Exosomal makers for pooled populations**

1. CD9/CD63/CD81/β-actin

**Exosomal makers for each population**

1. CD9

2. CD63

3. CD81

4. TCE

**Proteinase K treatment of exosomes**

1. MMP-2/TCE

2. - MMP-9/TCE

3. IL-6/TCE

**IL-6 signalling pathway**

1. Phospho-STAT3

2. β-actin

3. STAT3
