# Supplementary material for: Tridimensional cell culture of dermal fibroblasts promotes exosome-mediated secretion of extracellular matrix proteins
Source: Sci Rep. 2022 Nov 17;12:19786. doi: 10.1038/s41598-022-23433-0 (PMC9672399; doi:10.1038/s41598-022-23433-0)
Supplement: Supplementary file 2 — Supplementary Information 1. [file 41598_2022_23433_MOESM2_ESM.docx]

Supplementary Methods

**Exosomes uptake by dermal fibroblasts**

Fibroblasts were seeded at a concentration of 25,000 cells/well in 6-well plates containing sterile coverslips (Bellco glass, Vineland, NJ, USA) for 48 hours. They were rinsed 3 times with sterile PBS and once with DMEM. Then, 150 µg of exosomes per well, isolated from 3D cultures, were labelled using the PKH26 Red Fluorescent cell linker Kit (Sigma-Aldrich) following manufacturer’s instructions. Exosomes were then mixed with DMEM supplemented with 10% of exosome-depleted bovine FBS and added to fibroblasts for 16 hours. Wells were either treated with one of these antibodies: annexin A1, annexin A2, annexin V or annexin VI (0,18 µg/ml, Abcam). The coverslips were then fixed in paraformaldehyde 4% (PFA, EMS, Hatfield, PA, USA) for 30 minutes. After, we blocked in PBS-0.05% Triton- 5% goat serum for 1 hour at room temperature and labelled with Vimentin (1:1000; Abcam) primary antibody diluted in PBS-5% goat serum for 1 hour at room temperature. Cells were incubated for 30 minutes at room temperature in Alexa Fluor® 488-conjugated goat anti-rabbit (1:500; Invitrogen) then mounted using Fluoromount-G with DAPI (EMS). Samples were observed using a Zeiss Axio Imager M2 microscope equipped with an Axiocam HR Rev3 camera (Oberkochen, Germany).

**Mass spectrometry** (**nanoLC-MS/MS)**

Peptides were trapped at 20 μl/min in loading solvent (2% acetonitrile, 0.05% TFA) [BioBasic Inc., Markham, Ontario, Canada] on a 5 mm x 300 μm C18 pepmap cartridge pre-column (Thermo Fisher Scientific/Dionex Softron GmbH, Germering, Germany) for 5 minutes. The pre-column was then switched online with a self-made 50 cm x 75 µm internal diameter separation column packed with ReproSil-Pur C18-AQ 3-μm resin (Dr. Maisch HPLC GmbH, Ammerbuch-Entringen, Germany) and the peptides were eluted with a linear gradient from 5-40% solvent B (A: 0,1% formic acid, B: 80% acetonitrile, 0.1% formic acid) [BioBasic Inc., Markham, Ontario, Canada] for 90 minutes at 300 nL/min. Mass spectra were acquired using a data dependent acquisition mode using Thermo XCalibur software version 3.0.63. Full scan mass spectra (350 to 1800m/z) were acquired in the orbitrap using an AGC target of 4e5, a maximum injection time of 50 ms and a resolution of 120 000. Internal calibration using lock mass on the m/z 445.12003 siloxane ion was used. Each MS scan was followed by acquisition of fragmentation MSMS spectra of the most intense ions for a total cycle time of 3 seconds (top speed mode). The selected ions were isolated using the quadrupole analyser in a window of 1.6 m/z and fragmented by Higher Energy Collision-Induced Dissociation with 35% of collision energy. The resulting fragments were detected by the linear ion trap in rapid scan rate with an AGC target of 1e^4^ and a maximum injection time of 50 milliseconds. Dynamic exclusion of previously fragmented peptides was set for a period of 20 seconds and a tolerance of 10 ppm.

**Tube formation assay**

Human microvascular endothelial cells (HMVECs) were resuspended at a concentration of 1,000 cells/50 μl in Endothelial Cell Growth Medium MV2 additionned with the SupplementPack (PromoCell, Heidelberg, Germany) and 50 µg of exosomes isolated from 3D fibroblast cultures or the same proportion in volume of exosomes isolated from 3D fibroblast pre-treated with 30 μM GW4869 (Sigma-Aldrich). Then, 50 μl of the suspension mixture was added per well (96-wells plate) containing 50 μl of polymerized Growth Factor-Reduced Matrigel® (Corning Inc., Tewksbury, MA, USA) and the plate was incubated at 37°C for 24 hours. HMVECs were stained with 2 μg/mL Calcein-AM (Invitrogen) for 30 minutes and the enclosed networks capillary-like structure were imaged using an LSI 700 confocal microscope with Zeiss Axio Imager (Carl Zeiss Microscopy, Jena, Germany). Vessels-like tubes were analysed using the Image J software.

**Genomic DNA quantification**

Genomic DNA (gDNA) was extracted from cells using the DNAeasy blood and tissue kit (Qiagen). 2D samples, which contained fibroblasts harvested from 6 well plates, were incubated with proteinase K for 10 minutes at 56˚C (*N*=6, *n*=3), while 3D samples, which contained 12 mm punches (Acuderm Inc., Fort Lauderdale, FL, USA), were incubated overnight (*N*=6, *n*=3). The following steps were identical for both conditions and gDNA was eluted in 200 µl of UltraPure distilled water (Thermo Scientific). All gDNA samples concentration were measured twice using a NanoDrop 2000c (Thermo Scientific).

**Cytokine array**

The Proteome Profiler Human Cytokine Array kit (R&D Systems) was used for the quantification of exosomal cytokines, as described by the manufacturer’s protocol. Exosome samples, previously isolated from 1 ml of conditioned media, were first lysed with RIPA 1X (abcam) and resuspended in a total of 1 ml of PBS. Each membrane was incubated with 1 mL of lysed exosomes and the reconstituted detection antibody cocktail at 4°C overnight. Membranes were incubated with streptavidin-HRP followed by chemiluminescent detection reagents and signals were detected with a Fusion Fx7 imager (Vilber). Spots density was quantified with Image J software and normalised with the number of cells (gDNA) present in the culture dishes.

**ELISA against pro-collagen I alpha 1**

Pro-collagen 1α1 secretion was quantified using a specific Human Pro-collagen I alpha 1 ELISA kit (Abcam). All diluted supernatants were run in duplicate, and plates were read at a wavelength of 450 nm using a microplate reader (Bio-Rad).
